# Overcoming Radiation Resistance in Gliomas by Targeting Metabolism and DNA Repair Pathways

**DOI:** 10.3390/ijms23042246

**Published:** 2022-02-17

**Authors:** Wei Meng, Joshua D. Palmer, Michael Siedow, Saikh Jaharul Haque, Arnab Chakravarti

**Affiliations:** Department of Radiation Oncology, The Ohio State University, Columbus, OH 43210, USA; wei.meng@osumc.edu (W.M.); joshua.palmer@osumc.edu (J.D.P.); michael.siedow@osumc.edu (M.S.); saikh.haque@osumc.edu (S.J.H.)

**Keywords:** gliomas, radiation resistance, cancer metabolism, DNA repair

## Abstract

Gliomas represent a wide spectrum of brain tumors characterized by their high invasiveness, resistance to chemoradiotherapy, and both intratumoral and intertumoral heterogeneity. Recent advances in transomics studies revealed that enormous abnormalities exist in different biological layers of glioma cells, which include genetic/epigenetic alterations, RNA expressions, protein expression/modifications, and metabolic pathways, which provide opportunities for development of novel targeted therapeutic agents for gliomas. Metabolic reprogramming is one of the hallmarks of cancer cells, as well as one of the oldest fields in cancer biology research. Altered cancer cell metabolism not only provides energy and metabolites to support tumor growth, but also mediates the resistance of tumor cells to antitumor therapies. The interactions between cancer metabolism and DNA repair pathways, and the enhancement of radiotherapy sensitivity and assessment of radiation response by modulation of glioma metabolism are discussed herein.

## 1. Introduction/Background

Glioma is a broad category of central nervous system (CNS) tumors, which are one of the deadliest types of human cancers. Neomorphic mutations in isocitrate dehydrogenase (IDH) enzymes (arginine 132 for IDH1 and arginine 172 for IDH2) are found in 70% of gliomas and are highly associated with favorable outcomes of glioma patients [1]. By contrast, IDH1/2 wild-type glioma (IDHwt; without either of the mutations) is the most aggressive form of glioma, and IDHwt patients only have a short 6–12-month survival time [2]. Due to intratumoral heterogeneity and infiltrative nature, glioblastoma (GBM) is the most aggressive tumor, and it is notoriously resistant to chemoradiation therapy. The current standard of care for glioma patients largely relies on conventional temozolomide chemotherapy, and radiation has yielded only modest improvements in patient survival.

Cell metabolism provides the fundamental building blocks and energy sources for different cellular processes. Warburg’s study indicated that cancer cells can uptake a tremendous amount of glucose under normoxic conditions and produce lactic acid through aerobic glycolysis, the well-known Warburg effect [3]. Cancer cells gain proliferative advantages by increasing glucose uptake and aerobic glycolysis. Since then, there has been much interest in enhancing radiation sensitivity by modulating cell metabolism. Here, we review the interplay between cancer cell metabolism and DNA repair activities in brain tumors and the therapeutic potential of enhancing radiation sensitivity through cancer metabolic reprogramming.

## 2. Radiation Treatment and DNA Damage Responses

### 2.1. Radiation-Induced Cell Damages

Irradiation can cause DNA damage in mammalian cells, which was first shown in a microelectrophoretic study almost four decades ago [4]. It is important to understand the mechanisms by which ionizing radiation produces anti-tumor effects [5]. The major lesions induced by ionizing radiation are single-strand breaks (SSB) and double-strand breaks (DSB) in cellular DNA. Although DSBs arise less frequently than SSBs after radiation exposure, DSBs are arguably more lethal to cancer cells because DSB repair and G2/M checkpoint cannot guarantee genomic integrity and chromosomal stability. More recent significant finding is that radiation treatment produces so-called clustered DNA damages, multiple closely spaced DNA lesions (10–20 bp) on opposing strands [6].

In addition to DNA damage, radiation can also produce a variety of cellular perturbations and oxidative stress caused by reactive free radicals as one of the most significant consequences. In normal cellular metabolism, reactive oxygen species (ROS) are generated in aerobic cells, but cells can utilize multiple mechanisms to balance the redox homeostasis and eliminate oxidative stress such as glutathione (GSH), superoxide dismutase (SOD) and catalase, etc [7]. However, in many tumors and cancer cell lines, these protective mechanisms can be either shifted or made incapable of eliminating ROS generated from the actively proliferating cancer cells. For radiation treatment of cancer, the cellular redox system may be a critical determinant to enhance cancer cell killing while protecting normal tissues because ROS can oxidize cellular biomolecules such as proteins and lipids and activate many pathological processes [8]. While this mechanism has largely remained uncharted, ROS generation can become one of the primary weapons of radiotherapy for radiation-induced death of cancer cells [9].

Due to the complexity of the cellular damages induced by radiation treatment, the biological systems have a limited ability to repair these damages, which provides the theoretical and experimental foundations of radiation treatment [10].

### 2.2. DNA Damage Sensors and Early Responders

Cells harbor a cascade of proteins to recognize and repair exogenous and endogenous DSBs. One of the early responders sensing DSBs is the histone variant H2AX, which is rapidly phosphorylated on the Serine 139 residue after chemo- or radiation treatment. The number of γH2AX foci shows specific temporal and spatial relationships with cellular DNA damages [11]. The MRN complex (Mre11, Rad50, and Nbs1) can be independent from the H2AX-mediated DNA repair-signaling pathway by recruiting 53BP1 and BRCA1 to DSB sites and promote homologous recombination (HR) pathway [12]. Loss of MRN complex member (Mre11, Rad50, and Nbs1) can significantly sensitize the radiation treatment. Ku70/Ku80 heterodimer is composed of 70-kDa (Ku70) and 80-kDa (Ku80) subunits. In combination with DNA-dependent protein kinase (DNA-PK), Ku70/Ku80 form a multisubunit DNA repair machinery to repair DSBs through Non-homologous end joining (NHEJ) pathway [13,14]. It has been demonstrated that Ku70/Ku80 heterodimer’s DNA end-binding activity can dictate the radiosensitivity in human glioma, cervical cancer, and leukemia [15,16,17].

These DNA damage sensing proteins usually respond to radiation exposure in a few seconds to minutes range. The highly orchestrated crosstalk of the DNA damage sensing mechanisms is important to pass the signals between upstream or downstream proteins, whereby the radiation-damaged cells can engage subsequent repair pathways to maintain genomic integrity and chromosomal stability.

### 2.3. DNA Repair Pathways

Base excision repair (BER) and Nucleotide excision repair (NER) are the two key repair mechanisms of single-strand DNA damages. Several glycosylases can remove the ionizing radiation-induced DNA damage from the double-stranded helix to create apurinic or apyrimidinic (AP) site, then DNA polymerase fills the damaged sequence using a homologous template, and is subsequently sealed by DNA ligase. BER is a cell cycle dependent process [18,19]. For example, human apurinic/apyrimidinic endonuclease/redox factor-1 (APE1/Ref-1) is a dual function protein functioning as the major AP endonuclease in BER pathway as well as in the transcriptional regulation. The expression of human APE1 increases after the G1-S transition and peaks at the S phase, which has a direct impact on the cytotoxic effects of radiation and temozolomide treatment in glioblastoma cells [20,21,22]. NER removes the chemo- or radiation-induced bulky DNA lesions which is a multicomponent and multistep biological process. A 24 to 32 nucleotide-long oligomer is excised by endonuclease XPG (ERCC5).Then, polymerase and replication proteins fill in the gap followed by ligation [23]. DNA excision repair protein ERCC2 and ERCC1 are important components of the NER pathway. The polymorphism of ERCC1 and ERCC2 genes showed a significant association with the incidence of primary malignant brain tumors in a meta-analysis study [24].

There are two important pathways coordinating the repair of radiation-induced DSBs in mammalian cells: homologous recombination (HR) and non-homologous end-joining (NHEJ). Homologous recombination repair utilizes an undamaged DNA copy to guide the re-synthesis of the damaged DNA strand, which a repair process with high fidelity and takes place during the S and G2 phases of the cell cycle. On the other hand, NHEJ simply rejoins the broken DNA ends without guidance from a homologous template. The advantage of NHEJ is that this DSB repair mechanism is not limited to a specific phase of the cell cycle, but NHEJ is prone to lose DNA sequences at DSB sites [25]. Once the MRN complex is activated, ataxia-telangiectasia (ATM) kinase is recruited to trigger the DSB-signaling cascade and coordinate DNA repair processes. Mutations in ATM can notably impair HR and NHEJ pathways, which are crucial to repairing DNA damage caused directly by ionizing radiation (IR) or reactive chemicals [26]. Furthermore, ATM mutations confer enhanced radiation sensitivity and potentially develop late toxicity from cancer radiotherapy [27].

Cellular levels of metabolites are essential for the cells to recover from DNA damages. 5′ AMP-activated protein kinase (AMPK) is a highly conserved metabolic stress sensor protein which is phosphorylated by ATM in response to chemo- and radiotherapy-induced DNA damages in cancer cells [28]. The intracellular ATP level needs to be maintained to conduct the repairing processes of DNA damages, responding to the increased ATP consumption. The active AMPK can downregulate ATP-consuming biosynthetic processes and upregulate ATP-producing pathways to balance energy consumption and genomic integrity [29].

**Figure 1:**
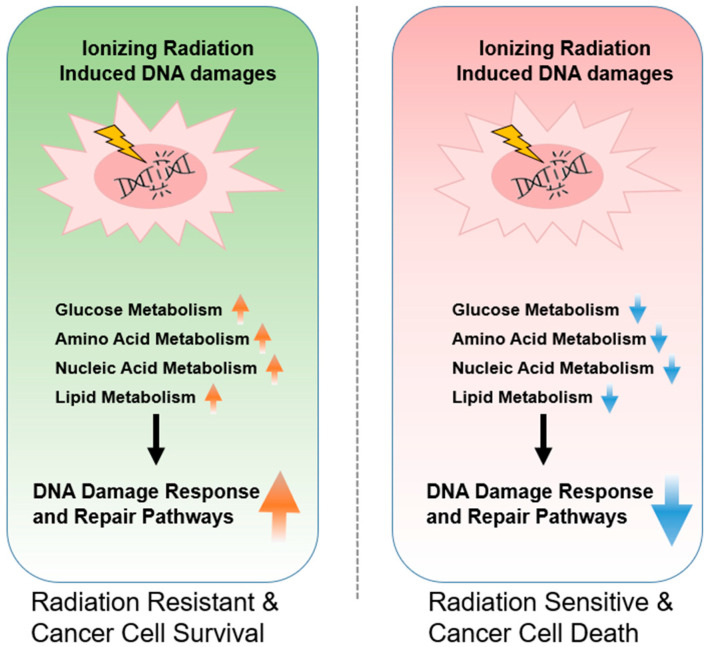
Radiation-induced DNA damage, repair mechanisms, and their potential relationships with cancer cell metabolism consequences in mammalian cells.

The overall mechanisms underlying the tumor metabolism related to radioresistance have illustrated in the Figure 1.

## 3. Preclinical Studies in Cancer Metabolism, Radiation Resistance, and DNA Repair Pathways

### 3.1. Glucose Metabolism and DNA Repair Pathways

In all normal cells, glucose is the primary source for energy generation. Glucose enters into cells via glucose transporters (GLUT), then gets phosphorylated by hexokinase (HK) to form glucose 6-phosphate. After undergoing a cascade of the enzymatic reactions, the six-carbon glucose is converted into two three-carbon pyruvate molecules. Under aerobic settings, pyruvate enters the tricarboxylic acid (TCA) cycle and is subsequently oxidized to carbon dioxide and water by mitochondrial enzymes to produce a maximum number of ATP molecules (38 ATPs per glucose). Under an anaerobic setting, the TCA cycle is not active, and glycolysis becomes the only option that cells can have for the production of ATP from glucose. This process is not very efficient and only produces 6–8 ATPs per glucose molecule, and the end product is lactic acid which is the main cause of exercise-induced muscle fatigue. Here we review the key enzymes found to be essential for the uptake of glucose in cancer cells and their roles in radiation resistance.

#### 3.1.1. Isocitrate Dehydrogenase

The three isocitrate dehydrogenase (IDHs) isoforms have a prominent place in cellular metabolism, which catalyzes the reversible oxidative decarboxylation reaction converting isocitrate to α-ketoglutarate to generate NADPH from NADP+. However, only mutations in IDH1 and 2 are considered to be the drivers of tumor initiation [30]. IDH1 R132 or IDH2 R172 mutant gliomas usually arise in a younger patient population, and patients with low grade gliomas (LGG) typically have a long overall survival time (5–10 years). On the other hand, IDH1 wild-type glioma is the most aggressive form of glioma, and IDHwt patients only have a short survival time (6–12-months) [2].

Mutant IDH1 proteins gain a new enzymatic activity at the cellular level compared to wild-type IDH proteins, leading to accumulation of oncometabolite d-2-hydroxyglutarate (2HG) in cancer cells. 2HG causes global accumulation of cytosine and histone methylations and drives chromatin to a closed structure, which reduces the activities of oncogenic signaling pathways [31]. Mutations in IDH1 and IDH2 have frequently been found in acute myeloid leukemia patients, 7% for IDH1 and 14% for IDH2, separately [32]. IDH2 R140 patients usually have a favorable outcome, however IDH2 R172 patients had a poor outcome suggesting these two mutations drive tumorigenesis in opposite directions [33].

#### 3.1.2. Pyruvate Dehydrogenase

Pyruvate dehydrogenase (PDH) oxidizes pyruvate to form the two-carbon unit acetyl-CoA which joins the metabolic pathways between glycolysis and TCA cycle. Pyruvate dehydrogenase exerts its functions by forming a large complex with dihydrolipoyl transacetylase and dihydrolipoyl dehydrogenase. It has been demonstrated that PDH component X (PDHX) is an essential gene for the cell growth of esophageal squamous cell carcinoma (ESCC) through metabolic regulations. The upregulated PDH function can be attributed to the co-amplification of PDHX and CD44 genes which are known markers of cancer stem cells in several different malignancies. Small molecule PDH inhibitors have been proven effective in pre-clinical models. CPI-613, a PDH inhibitor has been shown to inhibit the proliferation of cancer stem cells (CSCs) in vitro and the growth of ESCC xenograft tumors in vivo [34].

#### 3.1.3. Pyruvate Kinase

Pyruvate kinase is the last enzyme in glycolysis process, and its end-product, pyruvate, enters the TCA cycle to form ATP. There are four pyruvate kinase isoforms, but only Pyruvate Kinase Muscle 2 (PKM2) has been found to be oncogenic [35].

#### 3.1.4. Glucose Transporters

Glucose Transporter (GLUT) family proteins are encoded by the SLC2 genes and contain 14 members of the GLUT family in human, which actively transport monosaccharides across the lipid bilayers [36,37]. Over-expression of the facilitative GLUT1 protein has been observed in a large array of human cancer types suggesting that it likely plays a role in tumor initiation, progression and modulation of tumor immune microenvironment (TME) [38,39,40]. Radiation therapy can induce GLUT1 expression and upregulate glucose metabolism in MDA-MB-231 and MCF-7 breast cancer cells. Thus, inhibition of GLUT1 may enhance the radiation sensitivity through increasing ROS production. The transient inhibition of GLUT1 in laryngeal CSCs can increase radiosensitivity by downregulating the levels of RAD51 and DNA-PKcs [41]. Cancer cells treated with WZB117, a GLUT1 inhibitor, display downregulation of GLUT1 expression and glucose uptake. A combination of radiation therapy and a GLUT1 inhibitor demonstrated synergistic inhibition effect in the breast cancer cells [42]. GLUT1 inhibition not only alters the tumor metabolism, but also influences the tumor TME. Tumor-associated neutrophils (TANs) also rely on GLUT1 and glucose metabolism to support tumor growth.

#### 3.1.5. Hexokinase

Hexokinase (HK) phosphorylates six-carbon glucose to glucose-6-phosphate, which is the first enzymatic reaction in glycolysis. One glucose analog, 2-Deoxy-glucose (2-DG), can also be phosphorylated by hexokinase to form 2-deoxy-glucose-6-phosphate. However, this compound cannot be further processed by the enzymes in cells providing an opportunity to block glucose uptake. The addition of dietary 2-DG alone has been shown to inhibit Lewis lung carcinoma and its associated lung metastases and radiation-induced angiogenesis in a mouse model [43].

#### 3.1.6. Succinate Dehydrogenase

Succinate dehydrogenase (SDH) converts succinate to fumarate in the TCA cycle facilitating electron transport in the respiratory chain complex. Despite the rare occurrence of SDH mutations in humans, mutations in any SDH subunits can damage SDH complex assembly [44]. Hereditary paraganglioma and pheochromocytoma syndrome (PGL/PCC) results from the mutations in SDH subunits revealed the relationship between mitochondrial deficiency and tumor initiation [45]. The significant consequence of loss of SDH function is the accumulation of succinate, like neomorphic production of d-2HG in IDH1 mutant cancer, which causes the hypermethylation of DNA and histone 3 [46,47].

#### 3.1.7. Fumarate Hydratase

Fumarate Hydratase (FH) catalyzes the formation of malate from fumarate and the gene encoding this enzyme is located on chromosome 1p43. Heterozygous germline mutations of the FH gene have been found to relate to inherited uterine fibroids, skin leiomyomata and papillary renal cell cancer [48]. As a key member of the TCA cycle, FH function is essential for cell proliferation and survival. FH-deficient cells accumulate intracellular fumarate, display hypermethylation phenotype, and exhibit the hypoxic gene expression profiles even under normoxic conditions [49,50].

#### 3.1.8. Crosstalk between Glucose Metabolism and DNA Repair Pathways

Genetic inhibition of GLUT1 expression in TANs can significantly alleviate tumor growth and increase radiotherapy efficacy [38].

Increased radiosensitization of IDH1 mutant glioma cells was found in in vitro models through increasing apoptosis and upregulating ROS generation [51]. The crosstalk between DNA damage response and IDH1 mutation in the context of TP53 and ATRX loss indicated that IDH1 R132H acts as a tumor suppressor in glioma by epigenetically upregulating the ATM signaling pathway in mouse model and patient-derived xenograft model [52]. However, clinical trial suggested that IDH1 mutation glioma patients appeared to be sensitive to conventional chemoradiation therapy [53]. The possible reason is that 2HG can inhibit several DNA repair enzymes [54,55]. PDH complex plays an important role in citric acid cycle and aerobic glycolysis. A recent study suggested that reduced PDH activity can promote cancer cells to repair DNA damages through mitochondrial retrograde signaling [56]. It has recently been found that PKM2 is highly expressed in cancer cells and reshapes cancer metabolic programming. Phosphorylation of PKM2 at T328 by ATM, a key member of the DNA repair pathway, attenuates the response to ionizing radiation [57].

2-DG enhancing cytotoxicity and radiosensitization in tumor cells does not only depend on the glucose metabolism, but also decreases intracellular total glutathione content. The mechanism was demonstrated in the rescue experiment by adding thiol antioxidant *N*-acetylcysteine to the HeLa cells, which partially reversed the radiation-induced toxicity [58]. The results from the in vitro and in vivo models suggest that HK2, but not HK1 or HK3, determines the sensitivity of glioma cells to chemotherapy and/or radiation therapy through modulating the DNA damage responses [59].

Enhance radiation sensitivity was found in hamster B9 fibroblasts expressing a mutation in the gene coding for SDH subunit C. Disruption of mitochondrial metabolism can result in the elevation of intracellular ROS levels, which can dictate the biological effects of radiation [60].

One of the historical principles of radiation therapy and DNA-damaging chemotherapy is the accumulation of DNA damage and the resulting progression toward genomic instability. [61] One recently published study shed light on FH as a DNA repair factor in NHEJ by producing fumarate locally. FH-deficient cells gain resistance to the radiation-induced DNA damage by promoting rapid mitotic entry and fumarate-dependent promotion of NHEJ [62,63].

### 3.2. Amino Acid Metabolism and DNA Repair Pathways

#### 3.2.1. Glutamine Synthetase

Glutamine is also recognized as a non-essential amino acid because the magnesium-dependent glutamine synthetase (GS) catalyzes the reaction by condensing glutamate and ammonia to form glutamine. Cancer cells with specific genetic alterations such as MYC amplification can reprogram mitochondrial metabolism and upregulate glutaminolysis to support the production of energy and building blocks for cell division. The phenomenon can make cancer cells addicted to glutamine as their bioenergetic resource [64]. The recently developed glutamine antagonist 6-diazo-5-oxo-L-norleucine (DON, also termed JHU-083) can simultaneously suppress glycolysis and oxidative phosphorylation in cancer cells, which enhanced anticancer immune responses in a preclinical model. This study revealed metabolic checkpoint inhibitors as a promising and undefined field which may have a similar impact and immunotherapy with further investigation [65]. To identify patients who will benefit from glutamine deprivation therapy, the synergy between glycolysis and glutaminase inhibition is being actively investigated [66].

#### 3.2.2. Phosphoglycerate Dehydrogenase

Serine is a non-essential amino acid that can be taken from the diet or synthesized de novo via the serine synthesis pathway. The serine synthesis pathway connects many metabolic pathways, including glycolysis, nucleotide synthesis and S-adenosyl methionine cycle. The serine synthesis pathway starts with the glycolytic intermediate 3-phosphoglycerate (3PG). Phosphoglycerate dehydrogenase (PHGDH) catalyzes 3PG to 3-phosphohydroxypyruvate (3PHP), then phosphoserine aminotransferase 1 (PSAT1) converts 3PHP into 3-phosphoserine (3PS). Lastly, phosphoserine phosphatase (PSPH) hydrolyzes 3PS to form serine [67].

It has also been demonstrated that the dependence of cancer cells on serine can result from the amplification of the serine synthesis pathway enzymes especially PHGDH encoded by the gene located in chromosome 1p [68,69]. Furthermore, increased serine synthesis activity may present as a therapeutic target in some cancers. This observation revealed that the byproducts of de novo serine synthesis are essential for breast cancer by supporting nucleotide synthesis and maintaining cellular redox balance [69]. These findings suggest that targeting the enzymes involved in serine synthesis and limiting exogenous serine could be promising therapeutic approaches for cancer therapy.

NCT-502 and NCT-503 are two small-molecule inhibitors of PHGDH enzyme developed by David Sabatini’s group. Recent data have demonstrated that the biological or chemical inhibition of PHGDH reduces the incorporation of glucose-derived and exogenous serine into nucleotide synthesis by decreasing the activity of cytosolic serine hydroxymethyl transferase (SHMT1). NCT-503 displayed its ability to reduce the tumor volume of cell lines with high serine synthesis activity in the in vitro and orthotopic xenograft models [70]. CBR-5884 is another recently developed small molecular inhibitor of PHGDH, which shows a high selective toxicity to PHGDH-dependent tumor cells. This compound is a noncompetitive inhibitor that shows a time-dependent onset of inhibition and attenuates the PHGDH enzymatic activity on its oligomeric state [71].

#### 3.2.3. Argininosuccinate Synthetase and Argininosuccinate Lyase

Citrulline derived from the urea cycle is the precursor for arginine biosynthesis. Arginine, a non-essential amino acid, can be readily acquired from daily food consumption or de novo synthesized through the urea cycle in the normal cells by argininosuccinate synthetase (ASS) and argininosuccinate lyase (ASL) [72]. The most aggressive malignant tumors are usually associated with increased metabolic activities to support their high proliferation rate, metastasis, and invasion. Because various amino acids within cell survival pathways, arginine deprivation therapy becomes intriguing in arginine auxotrophic tumor types [73].

#### 3.2.4. Arginase

Arginine is an amino acid essential for T cell and natural killer (NK) cell proliferation. Two distinct isoforms, arginase (ARG) I and II, are located in the cytosol and in mitochondria, respectively [74]. Tumor-infiltrating myeloid cells expressing high levels of arginase can quickly deplete arginine in the tumor TME and promote immune evasion. CB-1158 is a recently developed small-molecule inhibitor of arginase that reverses the myeloid cell-mediated suppression of T cell functions. In vitro and syngeneic mouse models revealed that CB-1158 augmented tumor-infiltrating T cells and NK cells and delayed the tumor growth as a single agent or combined with checkpoint blockade therapy [75]. It is worth noting that 25 Gy irradiation induced mRNA and protein expression of arginase in the syngenic murine prostate cancer model, and, furthermore, tumor-associated macrophages (TAM) were found to be the sources of the elevated arginase expression [76]. It is known that radiation therapy can promote adaptive immune responses by the release of damage-associated molecular pattern (DAMP) molecules. Similarly, Tumor-associated myeloid cells restrain the adaptive immune response after radiation therapy through overexpressing arginase. Myeloid cell-specific ARG deletion in an in vivo model demonstrated an enhanced tumor control after radiation therapy [77].

#### 3.2.5. Crosstalk between Amino Acid Metabolism and DNA Repair Pathways

Glutamine deprivation or pharmacological inhibition of glutaminase can significantly promote radiation-induced apoptosis and sensitized pancreatic CSCs to fractionated radiation [78]. In addition, the in vitro and xenograft lung cancer models suggest a 30% increase in radiosensitivity after being treated with GLS inhibitors [79]. After examining ^13^Carbon metabolism in the GBM tumors and adjacent tissues of an orthotopic mouse model; the study indicated strikingly that a large accumulation of glutamine was found in the tumor tissues, which can only be partially explained by GS enzyme activity [80]. Several studies have demonstrated that the high glutamine synthetase activity is linked with the radiation resistance of cancer cells by modulating nucleotide metabolism, G2/M recovery, and DNA repair [81,82]. Moreover, high expression of glutamine synthase can support the proliferation of cancer cells in a novel non-canonical glutamine-independent pathway that, in turn, augment chemoradiation resistance in many cancers [79].

Some cancer cells have demonstrated increased de novo serine synthesis through upregulation of PHGDH indicating their survival depends on the sustainable supply of serine [83]. To the best of our knowledge, the available data investigating the efficacy of the radiotherapy combined with inhibition of PHGDH is very limited.

ASS is the limiting step of arginine biosynthesis, therefore, some pancreatic, skin, liver, and renal tumors that usually have low expression of ASS have demonstrated the enhanced sensitivity to arginine deprivation therapy in vitro and in vivo [84]. Arginine starvation has been associated with DNA damages and genome instability in breast cancer cells because low arginine level can reduce dNTP pools and affect DNA repair [85].

### 3.3. Nucleic Acid Metabolism and DNA Repair Pathways

Nucleotide synthesis and DNA replication are the basic requirements for cell proliferation, especially in the case of rapidly proliferating cancer cells. The biosynthesis of purine, including a six-membered and a five-membered nitrogen-containing ring, and pyrimidine, including a six-membered nitrogen-containing ring is critical for meeting this nucleotide demand. Furthermore, it provides the source of energy for driving cellular biological processes. There are two primary routes for the biosynthesis of nucleotides: de novo and salvage pathways [86]. Although the salvage pathway can recycle free purines and pyrimidines for nucleoside and nucleotide biosynthesis, most proliferating cells, especially cancer cells, synthesize nucleotides and nucleic acids de novo [87]. Understanding the regulation of the nucleotide salvage pathways by cancer cells can be vital to develop new strategies to target cancer cell proliferation using chemoradiation therapy.

#### 3.3.1. Phosphoribosyl Pyrophosphate Amidotransferase

The de novo pathway enzymes synthesize purine nucleotides from 5-phosphoribosyl-1-pyrophosphate (PRPP) with other simple molecules such as amino acids and tetrahydrofolate. The de novo purine metabolism demands high energy input such as ATP or GTP, which are required in five of the 12 sequential purine biosynthesis reactions [88]. Purine analogs 6-mercaptopurine (6-MP), 6-thioguanine (6-TG) and 8-Azaguanine (8-AG) were first developed to inhibit several enzymes involved in purine biosynthesis, including PRPP amidotransferase (PPAT). PPAT is the first the rate-limiting step of de novo purine synthesis [89].

#### 3.3.2. Inosine-5′-Monophosphate Dehydrogenase

Inosine-5′-monophosphate dehydrogenase (IMPDH) is a key enzyme which is involved in the de novo synthesis of guanine nucleotides. Additionally, IMPDH is also involved in the GTP salvage pathway though recycling the hypoxanthine. It has been demonstrated that the IMPDH-dependent de novo synthesis of guanine nucleotides is critical for radiation resistance of glioblastoma cells. Mycophenolic acid (MPA), preventing the formation of guanosine monophosphate, can deplete GTP concentrations and sensitize glioblastoma cells to radiation treatment in a dose-dependent fashion [90,91].

#### 3.3.3. Thymidylate Kinase

De novo pyrimidine nucleotide biosynthesis starts from carbamoyl phosphate, which is subsequently converted to uridine mono-, di-, and triphosphate (UMP, UDP, and UTP). These metabolites are the starting materials for de novo thymine nucleotide synthesis. The fundamental inhibition principles of purine and pyrimidine antimetabolites are similar. Cellular enzymes of the pyrimidine metabolic pathway partially convert the pyrimidine analogs, but these metabolites impair the functions of one or more downstream enzymes critical in DNA synthesis [92]. Recent findings indicated that thymidylate kinase (TMPK) is needed to elevate dNTPs pools in the vicinity of DNA damages, which can be determinant for the repair of DSBs [93].

#### 3.3.4. Thymidylate Synthase

Thymidylate synthase (TS) activity is the de novo source of thymidylate for DNA synthesis as part of the folate cycle, which is a valuable target for antimetabolites drug development. Fluorouracil (5-FU) which inhibits thymidylate synthase, is an analog of pyrimidine nucleoside [94]. 5-FU has been widely used to treat multiple solid tumors, including breast, prostate, colon, and cholangiocarcinoma cancers [95].

#### 3.3.5. Antifolate Therapy and Other Nucleotide Inhibitors

Antifolates were the first type of antimetabolites to achieve clinical success, more than 70 years ago. Antifolates act by disrupting one-carbon moieties supplied by the folate (vitamin B-9). The folate cycle is tightly connected to de novo purine and pyrimidine nucleotide metabolism. The mammalian target of rapamycin (mTOR) regulates a number of cellular processes, including cell survival, proliferation, and metabolism, and activation of mTOR signaling pathways is associated with human cancer [96]. It has been found recently that mTORC1 protein kinase complex induces purine synthesis by upregulating transcription factor ATF4, further augmenting the level of the methylenetetrahydrofolate dehydrogenase 2. Serial events lead to rewiring the anabolic purine synthesis pathways in cancer cells and supply the required precursor metabolites for cancer cell the growth with availability of required precursor metabolites [97]. Methotrexate (MTX), formerly known as amethopterin, inhibits de novo purine synthesis in human breast cancer cells by blocking dihydrofolate reductase reaction [88]. It has been known that dihydrofolate reductase (inhibited by methotrexate) and thymidylate synthase (inhibited by pemetrexed) are two key members of the folate cycle. Antifolate therapy has cured many cancer patients over the years because folates are essential for the synthesis of DNA and other nucleic acid molecules [98]. Drawbacks remain, however, as it has been reported that patients treated with a combination of MTX and ionizing radiation can cause a diversity of clinical syndromes, including neurotoxicities, as seen in children with acute lymphoblastic leukemia [99].

#### 3.3.6. Crosstalk between Nucleic Acid Metabolism and DNA Repair Pathways

The development of resistance in tumor cells toward pyrimidine and purine antimetabolite therapy generally results from an evolutionary process found in heterogenous tumor cell populations as they undergo the stress of cytotoxic therapies. In addition to the de novo purine and pyrimidine synthesis, the salvage pathway can also recycle free purines and pyrimidines for nucleoside and nucleotide biosynthesis. Understanding the regulation of the nucleotide salvage pathways by cancer cells can be vital to unfold new strategies to target cancer cell proliferation.

DNA damages demand cells to rewire their metabolic pathways to synthesize dNTP pool for better DNA repair abilities and a survival advantage. Glioma is one of the solid tumors exhibiting pronounced intratumoral genomic heterogeneity, therefore, inhibition of purine and pyrimidine nucleotide metabolism is an effective approach independent of genotype. The upregulated enzyme of de novo GTP synthesis is associated with lower survival rate in GBM patients [100]. Therefore, pharmacological inhibition of GTP synthesis can substantially sensitize radiation-resistant glioma cells. It was further demonstrated that MYC takes over the control of purine synthesis by reprogramming metabolisms of brain tumor initiating cells [101].

### 3.4. Lipid Metabolism and Radiation Sensitivity

A vast number of water-soluble and lipid-soluble metabolites circulate in the human body. A mass-spectrometry-based strategy researchers recently identified a specific serum lipidomic biosignature found in mice after ionizing radiation [102]. One particular feature of cancer cells is that they are able to reprogram their metabolic pathways to supply their increasing demand for energy as well as building blocks. The rewired cellular metabolism not only promotes tumor growth, but also contributes to the tumor-associated biological and clinical features such as invasion, migration, and resistance to treatments [103]. In recent years, novel metabolic targets have been proposed and studied in various cancers. Given fatty acids (FAs) are indispensable components in maintaining cellular energy storage and cell membrane integrity, targeting the lipid biosynthesis or lipogenesis of cancer cells has become a new antineoplastic therapeutic strategy [104].

#### 3.4.1. Acetyl-CoA Carboxylase

Acetyl-CoA carboxylase (ACC) is a cytosolic enzyme that catalyzes the carboxylation of acetyl-CoA to malonyl-CoA. This reaction is the first rate-limiting step of de novo fatty acid biosynthesis. ACC occupies a critical position in lipid metabolism, and has, thus, becomes an attractive drug target for cancer therapy. It has been shown that ACC is essential for the growth and survival of non-small-cell lung cancer (NSCLC) cells. ND-646, an allosteric inhibitor of the ACC enzymes, can impair tumor growth in the xenograft and genetically engineered mouse models [105]. Furthermore, despite initially being used as a drug to treat obesity and diabetes, Soraphen A can be repurposed as an inhibitor of acetyl CoA carboxylase activity interfering with de novo lipogenesis and beta-oxidation [106]. Pharmacological blockade of acetyl-CoA carboxylase using soraphen A can reduce the mammosphere formation in a dose-dependent manner, diminish the expansion of stem cell population, and potentially overcome chemotherapy and radiation resistance of CSCs [107]. Although it is unclear if inhibition of ACC could magnify the radiation sensitivity, it has been suggested that obese tissues can facilitate tumor hypoxic TME, which potentially leads to resistance to radiation therapy [108].

#### 3.4.2. Fatty Acid Synthase

Fatty acid synthase (FASN) is the rate-limiting enzyme for endogenous de novo production of FAs, and a druggable lipogenic oncogene [109]. Normal cells usually have low levels of FASN mRNA and protein expression. However, upregulation of FASN represents a common phenotypic alteration in many cancers, which indicates that lipogenic oncogenes have indispensable roles in tumor growth and tumor survival [110]. FASN expression has been significantly associated with progression and outcome of several types of cancer such as colon, prostate, and soft tissue sarcomas [111,112,113]. Studies have demonstrated that Food and Drug Administration (FDA)-approved proton pump inhibitors can effectively inhibit FASN and sensitize breast cancer cells to the doxorubicin and ionizing radiation therapy in a large breast cancer patient cohort [114].

#### 3.4.3. Carnitine Palmitoyltransferase

Carnitine palmitoyltransferase 1A CPT1 (Carnitine palmitoyl transferase I) and CPT2 (Carnitine palmitoyl transferase II) are a pair of rate-limiting enzymes that mediate oxidation of long-chain fatty acids (FAO) in mitochondrial. Mutations in the *CPT1A* gene restrain the normal cells from converting long-chain fatty acids into energy. The in vitro experiments demonstrate that the PPAR coactivator-1α (PGC1α) binds to CCAAT/enhancer binding protein β (CEBPB) and increases FAO regulated by CPT1A in nasopharyngeal carcinoma (NPC) cells. The upregulation of the PGC1α/CEBPB/CPT1A/FAO signaling pathway confers the radiation resistance and is linked with poor outcome of patients with NPC [115].

#### 3.4.4. Crosstalk between Lipid Metabolism and DNA Repair Pathways

Healthy cells and cancer cells can utilize lipids from the bloodstream through the de novo lipogenesis, but it was discovered that almost esterified FAs in tumor cells were obtained through de novo synthesis [116]. The studies indicate that when cancer cells have limited access to dietary cholesterol and fatty acids, de novo lipogenesis is activated in cancer cells. The anti-proliferation effect of lipogenesis inhibitors can be augmented by limiting exogenous fatty acid supplementation [104,117]. Efforts have been made to understand the relationship between de novo lipid synthesis and exogenous lipids and determine their particular roles in chemo and radiation therapy resistance [118].

The ketogenic diet is a high-fat, low-carbohydrate diet that increases ketone bodies in the blood. An intracranial mouse model of malignant glioma suggests that animals receiving a ketogenic diet displayed elevated levels of β-hydroxybutyrate and enhanced the anti-tumor effect of radiation [119]. This study indicates that cellular lipid metabolic alterations induced through the ketogenic diet may be useful to treat human malignant gliomas. This area is still under investigation, and several clinical trials are studying the tolerability and efficacy of a ketogenic diet in patients with recurrent glioma. However, the ketogenic diet failed to increase the efficacy of reirradiation in patients with recurrent malignant gliomas [120,121].

It has been suggested that radiation-induced FASN expression is essential for glioma cell survival [122]. Accordingly, it has been hypothesized that the uptake of exogenous lipids can bypass the suppressed lipid biosynthesis caused by FASN inhibition. A recent study reported that both exogenous and endogenous cholesterol could promote the radioresistance of cancer cells in in vitro and in vivo models. In contrast, blocking sterol response element-binding protein 1 (SREBP1) and FASN signaling can enhance radiation-induced CRC cell death [123]. Cerulenin synthetic analog C75, an inhibitor of fatty acid synthase, combined with X-ray irradiation can enhance radiation-induced apoptosis and delay the spheroid growth of prostate carcinoma in vitro [115].

Furthermore, recent studies suggested that lung cancer cell sublines previously exposed to the fractionated radiation in vitro confer notable radioresistance compared to their parental cells. Chemical inhibition of carnitine palmitoyltransferase 1A (CPT1A) by Etomoxir can significantly enhance radiosensitivity by downregulating the DNA repair pathways promoting the survival of lung cancer cells exposed to the ionized radiation [124]. The roles of metabolic enzymes in the DNA repair pathways are summarized in Table 1.

### 3.5. Tumor Metabolism and Immune Microenvironment

It is worth noting that an ideal radiation sensitizer only enhances the cell killing in the irradiated tumors but does not display single agent toxicity on tumor and normal cells. Therefore, almost no chemotherapy agent can meet these criteria as a radiation sensitizer. These agents should be described as radiation modifiers accurately, which exhibit synergistic actions with radiation treatment [131,132]. Some of the TME factors considered as radiation modifiers are reviewed in this section.

#### 3.5.1. Hypoxia-Inducible Factor and Vascular Endothelial Growth Factor

Further work in this area led to the discovery of hypoxia-inducible factor 1 alpha (Hif1α) and vascular endothelial growth factor (VEGF) whose expressions in concert drive angiogenesis, energy metabolism, and tumor cell invasion. Hypoxia-inducible factor-1a (HIF-1a), overexpressed in advanced cancers, in part, due to aberrant vascularization, often directly or indirectly reprograms the cancer-associated fibroblasts, macrophages, and extracellular matrix. These non-tumorous cells in the TME are remodeled by hypoxia and HIFs, leading to their functions to promote cancer growth [133]. VEGFs and their receptors (VEGFRs) are important pro-angiogenic factors that regulate the formation of new blood vessels. The tumor VEGF mediates the angiogenic response of irradiated tumors and ameliorates the radiation resistance in different cancer types. Several classes of inhibitors have been designed to target the VEGF activity or the surface receptor function to reduce tumor-initiated angiogenesis [134]. Anti-VEGF monoclonal antibody bevacizumab, targeting all isoforms of VEGF-A, is the most widely tested anti-angiogenesis therapy [135]. Clinical studies have demonstrated that bevacizumab benefits patients with advanced colorectal cancer, cervical cancer, and renal cell carcinoma [136].

#### 3.5.2. Glioma Stem Cells

Not all cancer cells are created equal, and they are instead organized hierarchically in heterogeneous tumor populations. Cancer stem cells (CSC) are a subtle subpopulation within a tumor, which possess self-renewal and DNA repair capacities. These features give CSCs strong resistance to chemotherapy and radiation therapy. How to eradicate all remaining CSC after surgical resection of a neoplasm become the root of tumor recurrence and metastases.

#### 3.5.3. Interplay between Cancer Cells and the Immune System

Immune cells within the TME display altered tumor metabolism in response to radiation and immunotherapies likely contributing to treatment resistance and ultimately recurrent disease [137]. In response to these cellular stresses, T cells, macrophages, and other components of the adaptive and innate immune system produce altered levels of oxidative species (ROS), fatty acid metabolism, and mTOR expression [138]. Although precise drug delivery has been challenging thus far, these pathways remain promising actionable targets and are being actively investigated in an attempt to “reprogram” the TME following therapy [139]. Tumor initiating cell or glioma stem cell (GSC) metabolism has been studied and remains an active area of research since their discovery in 1997 [140]. Using functional molecular imaging targeting the metabolite 2-[(18)F]fluoro-2-deoxy-d-glucose researchers have demonstrated that GSCs display altered oxygen consumption, intracellular ATP-levels, lactate production, glucose metabolism, and pyruvate kinase expression (PKM1/PKM2) when compared to their progenitors [141]. Both the immune modulating signals as well as the cells comprising the TME remain promising avenues for future therapeutic targets and are worth further investigation by the neuro-oncology community. 

## 4. Clinical Studies Targeting Metabolism of Brain Tumors

**Figure 2:**
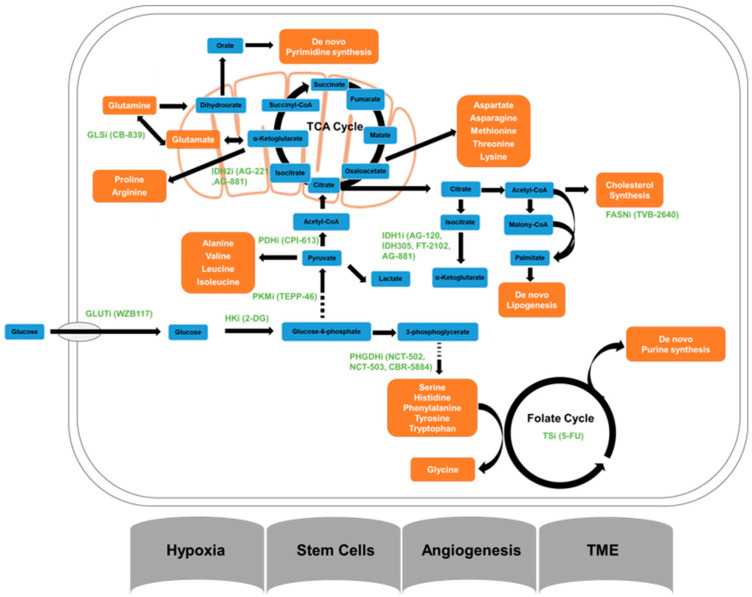
Major glioma metabolic pathways and inhibitors of metabolic pathways. FASNi, Fatty acid synthase; GLSi, glutaminase inhibitor; GLUT1, glucose transporter inhibitor; HKi, hexokinase inhibitor; IDH1i and IDH2i, isocitrate dehydrogenase 1 and isocitrate dehydrogenase 2 inhibitors; PDGDHi, Phosphoglycerate Dehydrogenase inhibitor; PDHi, pyruvate dehydrogenase inhibitor; PKMi, pyruvate kinase muscle inhibitor; TSi, Thymidylate synthase inhibitor.

All potential glioma metabolic pathways involved in the radioresistance are shown in Figure 2.

### 4.1. Targeting Glucose Metabolism

A post-hoc genomic analysis of RTOG 9802 supports the observations that patients with IDH-mutant glioma can benefit from chemoradiation therapy [142]. Several novel medicines targeting IDH mutant activity, such as ivosidenib (AG-120), IDH305, FT-2102, AG-221, and AG-881 are currently being tested in clinical trials. Preliminary phase I clinical data suggest that most of these IDH1 or 2 inhibitors are well tolerated, are associated with lower cellular 2-HG levels, and have potential antitumor activity, especially in IDH1-mutated gliomas. Phase I study NCT02073994 demonstrated that AG-120 with maximum tolerated dose is associated with favorable safety profile and prolonged tumor control in patients with advanced glioma [143]. Phase II study of IDH1 Inhibitor AG-120 in combination with nivolumab is being investigated in patients with IDH1 mutant gliomas. Studies to further evaluate the tumor control rate of IDH inhibitors with radiation in combination are ongoing [144,145]. Phase I/II trial NCT02273739 aims to determine the safety and maximum tolerable dose of AG-221 in the treatment of several types of IDH2 mutated solid tumors. The results from this multicenter trial have been released, but are not yet published in a peer-reviewed journal.

The administration of 2-DG to cancer patients has proved its efficacy in lowering blood glucose levels and reducing the glycolysis rate in the cancer patients; however, side effects exhibited include diaphoresis, flushing, drowsiness, and hypothermia, especially, are associated with 2-DG uptake [146]. The limited success of this inhibitor is like due to 2-DG-mediated alteration of energy production and radiation responses vary substantially in different cancer cell types [147].

Small molecule allosteric activator TEPP-46 stabilizes the tetrameric form of PKM2 and blocks PKM2 nuclear translocation, inhibiting tumor growth in a preclinical setting [148]. The synergistic effect of TEPP-46 and radiotherapy is remains to be explored.

Hexokinase II (HK2) protein expression has been shown to be elevated in GBM, which could serve as a useful therapeutic target [149]. The azole class of antifungal inhibitors that includes ketoconazole and posaconazole also displayed the antitumor effect on GBM in vitro in a small screening test. Two early phase I trials (NCT04869449 and NCT04825275) have been posted in early 2021 to establish the neuro-pharmacokinetic profiles of these HK2 inhibitors.

### 4.2. Targeting Amino Acid Metabolism

The mitochondrial enzyme glutaminases (GLS1 and GLS2) convert glutamine to glutamate; then glutamate is further converted to TCA cycle the substrate, alpha-ketoglutarate, via glutamate dehydrogenase (GLUD) [150]. To translate these findings to the clinic, a GLS-specific inhibitor CB-839 was developed to determine if cancer cells can gain response to chemotherapy or radiation treatment by suppressing glutaminolysis. CB-839 demonstrated antiproliferative activity in a triple-negative breast cancer (TNBC) cells and human epidermal growth factor receptor 2 (HER2)-positive breast cancer cells as a single agent or combined with paclitaxel [82]. The c-Myc-regulated amino acid transporter ASCT2 (SLC1A5) regulates the glutamine uptake in cancer cells. In combination with an ASCT2 inhibitor V-9302, the glutamine dependent cells can be sensitized to CB-839 treatment in several cancer types [151,152]. The ongoing phase Ib trial (NCT03528642) is investigating the glutaminase inhibitor CB-839 in combination with radiation therapy and temozolomide in treating patients with IDH-mutated diffuse or anaplastic astrocytoma.

Arginine, one of the common amino acids, has been linked to improving the immune system in people with brain tumors. A clinical trial has been completed to assess if the oral administration of arginine in powder form changes the immune function of GBM patients (NCT02017249). A pegylated form of prokaryotic arginine deiminase (ADI-PEG 20) has been investigated in phase I and II clinical trials and has been shown to catalyze the irreversible hydrolysis of arginine to citrulline and ammonia thus creating low cellular arginine levels [73]. A phase I study evaluating the safety and tolerability of ADI-PEG 20 in combination with radiotherapy and temozolomide in newly diagnosed GBM started recently (NCT04587830). The efficacy of this combination therapy is remains to be defined. PRT811 is a protein arginine N-methyltransferase 5 (PRMT5) inhibitor that exhibits the blood-brain barrier penetration. Although PRMT5 does not directly alter the arginine metabolism, it has been pursued as an alternative treatment choice because patients with high-grade gliomas have limited treatment options (NCT04089449).

### 4.3. Targeting Nucleic Acid Metabolism

Several clinical trials have tested to the combination of radiation therapy and radiation sensitizer/modifier 6-MP with adjuvant chemotherapy regimens in patients with primary malignant brain tumors. Although no increase in hematologic toxicity was observed, the addition of 6 MP did not demonstrate a significant survival benefit [153,154]. A phase II study evaluates the efficacy and safety of craniospinal radiation combined with 6-thioguanine, procarbazine, dibromodulcitol, lomustine, and vincristine (TPDCV) chemotherapy for treating pediatric astrocytoma and adult anaplastic ependymoma; the combination of TPDCV chemotherapy and radiation therapy does not differ substantially from radiation alone in with progression-free survival and overall survival [155].

Gemcitabine as a nucleoside analog currently is investigated in several solid tumors. Although gemcitabine has a modest ability to pass BBB, the concentration of gemcitabine in glioma cells is high enough for radiation sensitization [156]. Pre-irradiation gemcitabine chemotherapy demonstrated the safety profile but not effective in tumor control in phase II studies with newly diagnosed and recurrent GBM patients [157,158]. The phase I study evaluating gemcitabine concurrent with radiotherapy in patients with newly diagnosed malignant glioma yields promising outcomes [159].

### 4.4. Targeting Lipid Metabolism

BXQ-350 is a novel antitumor agent that suppresses glioma growth through interacting with the cancer cell membrane. BXQ-350 consists of two components, including the human lysosomal protein saposin C and the cell membrane phospholipid dioleoylphosphatidyl-serine (DOPS). The safety profiles and pharmacodynamics of BXQ-350 are being determined in several pediatric and adult brain tumors at the phase I stage (NCT04771897, NCT02859857, and NCT04404569).

A recent study indicated that abundant cholesterol supply is essential to sustain the progression of glioma cells [160]. Evolocumab is an FDA-approved proprotein convertase/kexin type 9 serine protease inhibitor for hypercholesterolemia treatment. To re-purpose this antibody inhibitor to cancer patients, NCT04937413 is a phase 0 study to determine the pharmacokinetics and pharmacodynamics of evolocumab in patients with high-grade glioma.

### 4.5. Targeting Immune Modulation

Hif-1α supports cancer cell survival under hypoxic conditions through its activation of nuclear factor kappa B (NF-κB) as well as promotion of expression of ligands for program death receptor 1 (PD-1) and cytotoxic T-lymphocyte-associated protein 4 (CTLA-4) [161]. To date, 31 trials have investigated inhibitors of the PD-1/PDL-1/CTLA-4 axis in high-grade gliomas. Checkmate-143 (NCT 02017717), a randomized phase III trial, found a small improvement in progression free survival, median overall survival, and overall response rate with the addition of ipilimumab to nivolumab versus nivolumab alone in GBM [162]. Checkmate-548 (NCT 02667587) is an actively accruing phase III clinical trial in newly diagnosed GBM patients with an unmethylated MGMT promoter; patients are randomized to temozolomide and radiation therapy or nivolumab and radiation. The phase 2 clinical trial NCT02968940 explored the hypofractionated radiation therapy combined with avelumab in adult GBM patients with IDH mutation. Avelumab is a fully humanized monoclonal antibody blocking the interaction between PD-L1 and PD-1 and restoring the cytotoxic T cell response. The trial has been completed; however, no results have been reported. Although PD-1/PDL-1 inhibitors haven’t had the same success as in NSCLC/SCLC, they remain a viable therapeutic option in this patient population which hasn’t seen significant progress since temozolomide was adopted as first line therapy in 2005 [163].

## 5. Metabolic Imaging for Assessment of Radiation Response for Glioma

### 5.1. Metabolites/Tracers and Guiding Radiation Planning/Retreatment

The most common and prevalent radiotracer is F-18-fluorodexyglucose (F18-FDG), which is a surrogate for metabolic activity, common in neoplastic processes. Unfortunately, F18-FDG PET demonstrates diffuse activity in the brain given its high metabolic demand, making the tumor to background ratio not ideal for differentiating tumor from normal brain. This is especially true when differentiating tumor recurrence from post-treatment changes, such as pseudoprogression and radiation necrosis.

Radiolabeled amino acids were introduced in the 1980s as positron emission tomography (PET) tracers for brain tumors [164], due to an increased amino acid utilization within glial tumors. Active amino acid transport in tumor cells is supposed to be one of the rate-limiting factors of amino acid imaging and is upregulated in tumor cell membranes [165,166]. This provides a high tumor to background ratio compared to normal brain tissue. Amino acid PET has demonstrated higher diagnostic accuracy when compared to MRI for glioma grading, differentiation of glioma from non-neoplastic lesions, and differentiation of glioma recurrence from treatment induced changes. Amino acid PET is superior to MRI for assessment of treatment response as well as delineation of glioma extent for enhancing and non-enhancing tumors.

In glioma, patients’ treatment-related effects, otherwise known as pseudoprogression or radiation necrosis, limits the reliability of conventional MRI to assess treatment response [167]. Several studies reported only a limited value of early post-radiotherapy quantitative FDG PET changes for the assessment of response to radiotherapy, either alone or with concomitant temozolomide [168,169]. Early changes of tumor-to-brain FET uptake ratios following chemoradiation with temozolomide in newly diagnosed GBM patients have been shown to be a strong predictor for progression-free and overall survival [170,171]. In contrast, changes in the volume of contrast enhancement on MRI were not associated with survival.

### 5.2. The Application of Metabolic Imaging in Clinical Trials

Development of [18 F] DASA-23 for imaging tumor glycolysis has been tested in the Phase 1 study (NCT03539731) to evaluate the pyruvate kinase M2 (PKM2) expression in patients with intracranial tumors or recurrent GBM as well as healthy volunteers by positron emission tomography (PET) scan technique. The primary goal of this study is to determine whether the [18F]DASA 23 PET scan can predict tumor’s responsiveness to antitumor therapy. This trial is currently recruiting new patients. Similarly, hyperpolarized carbon-13 pyruvate is also being tested in the NCT04019002 trial launched recently by the University of California, San Francisco.

Branched-chain amino acids (BCAAs) are essential amino acids, including valine, leucine, and isoleucine. The imbalanced BCAAs in serum is a physiological evidence of chronic liver diseases [172]. Tryptophan, a BCAA, was used as a radioactive tracer in the brain using PET scanning in an ongoing clinical trial (NCT02367482). The goal of this study is to determine if tryptophan metabolism is a useful approach to distinguish between different types of brain tumors. We summarized the selected agents targeting glioma metabolism that are evaluated in preclinical studies and clinical trials (Table 2).

## 6. Conclusions

Resistance to chemo- and radiotherapy is the decisive implication of cancer treatment failure. Recent advances in high-throughput technologies, have allowed us to classify gliomas into different categories based on their genetic and epigenetic lesions, instead of the histopathological classifications conventionally used by clinical pathologists. This systematic and molecular knowledge of glioma cells allows us to revisit many signaling pathways and complex interactions, including cancer metabolism and DNA repair activities. In glioma cells, intracellular glucose, lipid, amino acid, and nucleotide levels are dramatically upregulated through extracellular uptake, de novo synthesis, and other molecular mechanisms; in so doing, the metabolic reprogramming supports aggressive proliferation, progression, and chemoradiation resistance in gliomas. One of the resistance mechanisms is that aberrant glioma metabolism boosts the rapid repair of DNA lesions introduced by radiotherapies. Through decades of study, it has been proven that the strategy of genetic and pharmacological inhibition of glioma metabolism combined with radiotherapy has achieved limited success to different solid tumors, including glioma. However, it is necessary to consider the inter-and intra-tumoral heterogeneity and the immune TME because glioma cells can dodge the therapeutic stress (e.g., radiotherapy) through fostering tumor cell evolution and interacting with different TME cell populations. A better understanding of the role of these factors in metabolic reprogramming may help us develop novel therapeutic strategies in the future.

We recognize the following limitations. (1) Due to the lack of available information on the blood-brain barrier (BBB) permeability, we cannot truly predict the efficacy of these therapeutic agents alone or in combination with radiation therapy. However, these radiation and chemotherapy can also disrupt the structure of BBB, thereby allows the entry of these agents. (2) Many solid malignancies, including glioma, are treated with radiation therapy and/or chemotherapy that trigger the same or similar molecular pathways leading to the very similar outcome and, therefore, extend this review to other malignancies.

## Figures and Tables

**Table 1:** The metabolic enzymes involved in DNA repair pathways.

Metabolic Enzyme	Canonical Function	DNA Repair	References
Isocitrate dehydrogenase (IDH)	Conversion from isocitrate to α-ketoglutarate	HR	[52,55]
Pyruvate dehydrogenase (PDH)	Oxidization of pyruvate	NHEJ and HR	[56]
Pyruvate Kinase Muscle 2 (PKM2)	Pyruvate production	HR	[57]
Glucose Transporter (GLUT)	Transportation of monosaccharides across cell membrane	NHEJ and HR	[41,125]
Hexokinase (HK)	Phosphorylation of six-carbon glucose to glucose-6-phosphate	NHEJ and HR	[125]
Succinate dehydrogenase (SDH)	Oxidation of succinate to fumarate	HR	[126]
Fumarate Hydratase (FH)	Conversion of fumarate to malate	NHEJ and HR	[62,63]
Glutamine synthetase (GS)	Production of glutamine	HR	[78,81]
Argininosuccinate synthetase (ASS)	Synthesis of argininosuccinate from citrulline and aspartate	NHEJ	[127]
Arginase (ARG)	Hydrolysis of arginine to ornithine and urea	NHEJ	[128]
Thymidylate synthase (TS)	Production of Thymidylate	NHEJ	[129]
Fatty acid synthase (FASN)	Synthesis of fatty acid	NHEJ	[130]
Carnitine palmitoyltransferase 1A (CPT1)	Modulation of fatty acid beta-oxidation	No clear	[124]

**Table 2:** List of drugs and imaging techniques targeting glioma metabolism in clinical trial.

Target	Type	Drug	NCT Number	Status	Clinical Trial Phase	Combined with Radiotherapy
IDH1 and IDH2 mutant	Inhibitor	AG-120	NCT04056910	Recruiting	Phase II	N
			NCT03343197	Active, not recruiting	Phase I	N
			NCT02073994	Active, not recruiting	Phase I	N
			NCT04195555	Recruiting	Phase II	N
	Inhibitor	IDH305	NCT02381886	Active, not recruiting	Phase I	N
	Inhibitor	FT-2102	NCT03684811	Active, not recruiting	Phase I|Phase II	N
	Inhibitor	AG-221	NCT02273739	Completed	Phase I|Phase II	N
	Inhibitor	AG881	NCT03343197	Active, not recruiting	Phase I	N
			NCT02481154	Active, not recruiting	Phase I	N
			NCT04164901	Recruiting	Phase III	N
PDH	Inhibitor	CP-613	No trial	NA	NA	NA
PKM2	Inhibitor	TEPP-46	No trial	NA	NA	NA
GLUT1	Inhibitor	WZB117	No trial	NA	NA	NA
GLS1	Inhibitor	CB-839	NCT03528642	Recruiting	Phase I	Y
PHGDH	Inhibitor	NCT-502	No trial	NA	NA	NA
		NCT-503	No trial	NA	NA	NA
		CBR-5884	No trial	NA	NA	NA
Arginine Metabolism	Inhibitor	ADI-PEG 20	NCT02029690	Terminated	Phase I	N
		Oral Arginine	NCT02017249	Completed	Phase I	N
TS	Inhibitor	5-FU	NCT01498783	Completed	Phase II	N
FASN	Inhibitor	TVB-2640	NCT03032484	Active, not recruiting	Phase II	N
Lipid Metabolism	Inhibitor	BXQ-350	NCT02859857	Completed	Phase I	N
			NCT04404569	Recruiting	Phase I	N
Ketosis	Inhibitor	Ketogenic diet and metformin	NCT04691960	Recruiting	Phase II	N
Carbohydrate metabolism	Inhibitor	Metformin	NCT02149459	NA	Phase I	Y
Valine metabolism	Imaging	C13 N15 Valine	NCT02305056	Terminated	Phase I	N
Pyruvate metabolism	Imaging	Hyperpolarized Carbon C13 Pyruvate	NCT04540107	Recruiting	Phase I	N
Tryptophan metabolism	Imaging	Positron emission tomography	NCT02367482	Recruiting	NA	N
Lactate and other metabolites	Imaging	Magnetic Resonance Spectroscopy	NCT01138813	Completed	NA	N
Metabolic Tumor Volume	Imaging	Magnetic Resonance Spectroscopy	NCT02006563	Completed	NA	N
